# Effectiveness of Colonoscopy Screening vs Sigmoidoscopy Screening in Colorectal Cancer

**DOI:** 10.1001/jamanetworkopen.2024.0007

**Published:** 2024-02-29

**Authors:** Frederik E. Juul, Amanda J. Cross, Robert E. Schoen, Carlo Senore, Paul F. Pinsky, Eric A. Miller, Nereo Segnan, Kate Wooldrage, Paulina Wieszczy-Szczepanik, Paola Armaroli, Kjetil K. Garborg, Hans-Olov Adami, Geir Hoff, Mette Kalager, Michael Bretthauer, Øyvind Holme, Magnus Løberg

**Affiliations:** 1 Clinical Effectiveness Research Group, University of Oslo, Oslo, Norway; 2 Clinical Effectiveness Research Group, Oslo University Hospital, Oslo, Norway; 3 Cancer Screening & Prevention Research Group, Department of Surgery & Cancer, Imperial College London, London, United Kingdom; 4 Division of Gastroenterology, Hepatology and Nutrition, Department of Epidemiology, University of Pittsburgh, Pittsburgh, Pennsylvania; 5 University Hospital Città della Salute e della Scienza, Turin, Italy; 6 Division of Cancer Prevention, National Cancer Institute, Rockville, Maryland; 7 Department of Gastroenterology, Hepatology and Clinical Oncology, Centre of Postgraduate Medical Education, Warsaw, Poland; 8 Department of Medical Epidemiology and Biostatistics, Karolinska Institutet, Stockholm, Sweden; 9 Section for Colorectal Cancer Screening, Cancer Registry of Norway, Oslo, Norway; 10 Department of Research and Development, Telemark Hospital Trust, Skien, Norway; 11 Institute of Clinical Medicine, University of Oslo, Oslo, Norway; 12 Department of Medicine, Sorlandet Hospital Health Trust, Kristiansand, Norway

## Abstract

**Importance:**

Randomized clinical screening trials have shown that sigmoidoscopy screening reduces colorectal cancer (CRC) incidence and mortality. Colonoscopy has largely replaced sigmoidoscopy for CRC screening, but long-term results from randomized trials on colonoscopy screening are still lacking.

**Objective:**

To estimate the additional screening benefit of colonoscopy compared with sigmoidoscopy.

**Design, Setting, and Participants:**

This comparative effectiveness simulation study pooled data on 358 204 men and women randomly assigned to sigmoidoscopy screening or usual care in 4 randomized sigmoidoscopy screening trials conducted in Norway, Italy, the US, and UK with inclusion periods in the years 1993 to 2001. The primary analysis of the study was conducted from January 19 to December 30, 2021.

**Intervention:**

Invitation to endoscopic screening.

**Main Outcomes and Measures:**

Primary outcomes were CRC incidence and mortality. Using pooled 15-year follow-up data, colonoscopy screening effectiveness was estimated assuming that the efficacy of colonoscopy in the proximal colon was similar to that observed in the distal colon in the sigmoidoscopy screening trials. The simulation model was validated using data from Norwegian participants in a colonoscopy screening trial.

**Results:**

This analysis included 358 204 individuals (181 971 women [51%]) aged 55 to 64 years at inclusion with a median follow-up time ranging from 15 to 17 years. Compared with usual care, colonoscopy prevented an estimated 50 (95% CI, 42-58) CRC cases per 100 000 person-years, corresponding to 30% incidence reduction (rate ratio, 0.70 [95% CI, 0.66-0.75]), and prevented an estimated 15 (95% CI, 11-19) CRC deaths per 100 000 person-years, corresponding to 32% mortality reduction (rate ratio, 0.68 [95% CI, 0.61-0.76]). The additional benefit of colonoscopy screening compared with sigmoidoscopy was 12 (95% CI, 10-14) fewer CRC cases and 4 (95% CI, 3-5) fewer CRC deaths per 100 000 person-years, corresponding to percentage point reductions of 6.9 (95% CI, 6.0-7.9) for CRC incidence and 7.6 (95% CI, 5.7-9.6) for CRC mortality. The number needed to switch from sigmoidoscopy to colonoscopy screening was 560 (95% CI, 486-661) to prevent 1 CRC case and 1611 (95% CI, 1275-2188) to prevent 1 CRC death.

**Conclusions and Relevance:**

The findings of this comparative effectiveness study assessing long-term follow-up after CRC screening suggest that there was an additional preventive effect on CRC incidence and mortality associated with colonoscopy screening compared with sigmoidoscopy screening, but the additional preventive effect was less than what was achieved by introducing sigmoidoscopy screening where no screening existed. The results probably represent the upper limit of what may be achieved with colonoscopy screening compared with sigmoidoscopy screening.

## Key Points

**Question:**

What is the long-term additional benefit on colorectal cancer incidence and mortality associated with colonoscopy screening vs sigmoidoscopy screening?

**Findings:**

This comparative effectiveness simulation study of 358 204 adults showed a statistically significant 7 percentage point reduction in colorectal cancer incidence and mortality at 15-year follow-up of colonoscopy screening, which proportionally amounted to 30% benefit compared with sigmoidoscopy screening.

**Meaning:**

These findings suggest that the added benefit of colonoscopy screening is less than introducing sigmoidoscopy screening where no screening exists.

## Teaser

This comparative effectiveness study estimates the additional long-term screening benefit of colonoscopy compared with sigmoidoscopy on colorectal cancer incidence and mortality in adults.

## Introduction

Sigmoidoscopy and colonoscopy are recommended screening tests for colorectal cancer (CRC).^1,2^ In 4 large randomized clinical trials, sigmoidoscopy screening reduced CRC incidence and mortality predominantly in the distal colon and rectum.^3,4,5,6^ Colonoscopy has largely replaced sigmoidoscopy for CRC screening.^7,8^ Observational studies suggest that colonoscopy is also associated with reduced proximal colon cancer incidence and mortality.^9,10^ The Nordic-European Initiative on Colorectal Cancer (NordICC) randomized clinical trial presented follow-up data comparing colonoscopy screening with usual care; after a 10-year follow-up, colonoscopy screening reduced CRC incidence by 18%, while no statistically significant reduction was observed for CRC mortality.^11^ Data on the effect of colonoscopy screening after longer follow-up are lacking from randomized trials.^12^ We therefore used a simulation analysis to estimate the additional benefit of colonoscopy compared with sigmoidoscopy.

## Methods

### Included Trials

For this comparative effectiveness study, we pooled data from 4 randomized clinical trials with 15 or more years of follow-up after sigmoidoscopy screening for CRC from Norway (Norwegian Colorectal Cancer Prevention trial [NORCCAP]), the US (Prostate, Lung, Colorectal and Ovarian Cancer Screening Trial [PLCO]), the UK (UK Flexible Sigmoidoscopy Screening Trial [UKFSST]) and Italy (Screening for Colon Rectum trial [SCORE]).^3,4,5,6^ Inclusion periods were in the years 1993 to 2001. Furthermore, we used the colonoscopy screening results from the Norwegian part of the NordICC trial.^11^ All trials were approved by ethics committees in their countries or regions and are registered in clinical trial registries. All participants in the trials, except those randomly assigned to the control group of the NORCCAP trial (who were never contacted), provided written informed consent. Details on each endoscopy screening trial have been published previously and are described in brief in eMethods 1 in Supplement 1.^3,4,5,6^ The current study complies with the International Society for Pharmacoeconomics and Outcomes Research (ISPOR) reporting guideline for comparative effectiveness research (eAppendix 1 in Supplement 1).^13^

### Data Acquisition

We extracted aggregated data from the 4 sigmoidoscopy screening trial databases and merged them into a central database at the University of Oslo, Oslo, Norway. Due to the data protection legislation in Europe, individual patient data could not be shared across the trials (eMethods 2 in Supplement 1). Instead, we used aggregated anonymized data, including number of individuals aged 55 to 64 years in each trial, person-years of follow-up for incidence and mortality, and most recent number of CRC cases and deaths, by cancer site, randomization group, and sex.^3,4,5,6^ Individuals aged 50 to 54 years in NORCCAP and 65 to 74 years in the PLCO were not included in our analysis.

For individuals invited to a screening, we collected data on screening attendance and referral for colonoscopy after positive screening sigmoidoscopy,^3,4,5,6^ colonoscopy attendance, and details on polyps and CRCs detected at sigmoidoscopy screening and colonoscopy. For validation of our simulation, we used data from the Norwegian part of the NordICC colonoscopy screening trial on CRC incidence.^11^ We also used 10-year follow-up data from NORCCAP.^14^

### Simulation of Colonoscopy Screening

**Figure 1.**
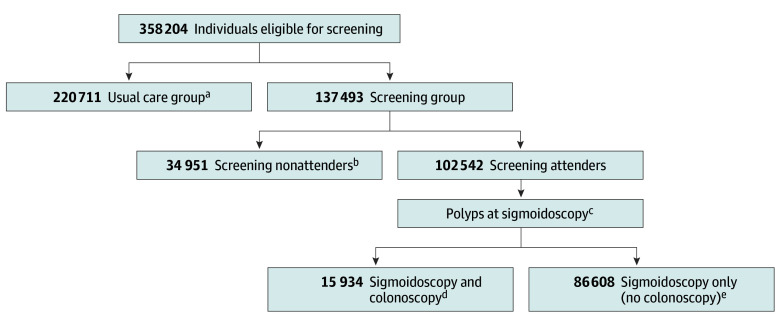
Flowchart of Sigmoidoscopy Screening as Applied in the 4 Trials All trial individuals were categorized into 4 groups depending on randomization, attendance, and sigmoidoscopy findings (resulting in colonoscopy or not): usual care, nonattenders, screening positive, and screening negative. ^a^Usual care group: individuals randomly assigned to usual care. ^b^Nonattenders group: individuals randomly assigned to sigmoidoscopy screening but declined to undergo screening. ^c^Trial criteria for referral to colonoscopy are described in eTable 1 in Supplement 1. ^d^Screening positive group: individuals randomly assigned to sigmoidoscopy screening, attended screening, and had a positive screening test leading to a subsequent colonoscopy. ^e^Screening negative group: individuals randomly assigned to sigmoidoscopy screening, attended screening, but did not have a subsequent colonoscopy. This group also included individuals with a positive sigmoidoscopy screening test who did not have a subsequent colonoscopy.

The observed effects of sigmoidoscopy screening in the 4 trials have been published.^3,4,5,6^ We estimated the effectiveness of colonoscopy screening for CRC incidence and mortality and compared the results with both usual care and actually performed sigmoidoscopy screening by simulation analyses applying the following assumptions:

- Attendance for screening colonoscopy is the same as the observed attendance for screening sigmoidoscopy in the trials.
- For participants who had a colonoscopy after a positive sigmoidoscopy, the effectiveness of screening colonoscopy (for CRC incidence and mortality) is the same in these individuals (Figure 1).
- The reduction in distal CRC incidence and mortality is the same after colonoscopy screening as the observed reduction after sigmoidoscopy screening (Figure 1).
- The relative reduction in proximal colon cancer incidence and mortality after colonoscopy screening is the same as the relative reduction in the distal colorectum observed in the sigmoidoscopy trials.

In all analyses, the distal colorectum was defined as the descending colon, sigmoid colon, and rectum. All other segments were defined as the proximal colon. Our colonoscopy screening simulation assumed a once-only screening invitation and probably represents an upper limit of what may be achieved with colonoscopy compared with sigmoidoscopy screening. We did not estimate adverse effects or harms of colonoscopy.

### Statistical Analysis

All trial participants were assigned 1 of 4 groups, as defined in Figure 1. In the PLCO, individuals were counted as attenders if they underwent screening sigmoidoscopy at baseline or 3 or 5 years after baseline. All analyses were performed for CRC incidence and CRC mortality, for women and men combined and separately.

#### Sigmoidoscopy Screening Effectiveness

First, we calculated the cumulative rates (events per 100 000 person-years) and risks (events per 100 000 individuals after a 15-year follow-up) for CRC incidence and CRC mortality for the sigmoidoscopy screening and usual care group in each trial, overall and by site (proximal colon and distal colorectum) (eMethods 3 in Supplement 1, step 1). Next, we used the cumulative incidence rates to calculate rate ratios and rate differences (cases prevented per 100 000 person-years) for sigmoidoscopy screening compared with usual care for the individual trials. Similarly, we used risks to calculate the risk difference (cases prevented per 100 000 individuals 15 years after screening). The number needed to invite to screening (NNS), to prevent 1 CRC case or 1 CRC death was calculated as the inverse of the risk difference. The individual trial results were then pooled to calculate overall cumulative rates, rate ratios, risks, risk differences, and NNS.

#### Colonoscopy Screening Effectiveness

To estimate the additional number of cases prevented with colonoscopy, we used the observed number of proximal colon cancer cases among screening attenders who did not have a colonoscopy (the screening negative group) (Figure 1) and the sigmoidoscopy rate ratios for distal CRC incidence (rate ratio sigmoidoscopy, distal colon) among all screening attenders. In each trial, the number of proximal CRC cases prevented by colonoscopy was calculated as the observed number of proximal cases among the screening negatives multiplied by 1 minus rate ratio sigmoidoscopy, distal colon (ie, proximal CRC cases prevented = observed number of proximal CRC cases in screening negative × [1 – rate ratio sigmoidoscopy, distal colon]) (Figure 1; eMethods 3 in Supplement 1, step 2). The resulting numbers of CRC cases prevented were then subtracted from the observed total number of CRC cases in the sigmoidoscopy screening group (which included the screening nonattenders group, sigmoidoscopy and colonoscopy group, and sigmoidoscopy only, no colonoscopy group (Figure 1) to estimate the number of CRC cases colonoscopy screening would capture (CRC cases prevented by colonoscopy = observed number of CRC cases in screening group – proximal CRC cases prevented [by switching to colonoscopy]). Next, we calculated cumulative rates, rate ratios, and rate differences following colonoscopy screening compared with usual care for the individual trials. The individual trial results were then pooled to calculate cumulative rates, rate ratios, rate differences, risks, risk differences, and NNS for colonoscopy screening across trials. A similar method was used to calculate number of CRC deaths prevented by colonoscopy.

#### Colonoscopy Screening vs Sigmoidoscopy Screening

We calculated rate ratios, rate differences, and risk differences for colonoscopy screening compared with sigmoidoscopy screening for the individual trials and pooled the results as described for sigmoidoscopy and colonoscopy screening effectiveness. The number of individuals needed to switch from sigmoidoscopy to colonoscopy screening to prevent 1 additional event was calculated as the inverse of the risk difference between sigmoidoscopy screening and colonoscopy screening.

#### Calculations

Trials were pooled with the inverse variance of trial specific estimates (eg, log rate ratio, rate difference) as weights, with the variance of the overall estimates calculated as the inverse of the sum of the weights. We used bootstrapping to estimate the variances of the colonoscopy screening effectiveness metrics (eg, log rate ratio, rate difference) in the individual trials, because their estimates, which involved the distal sigmoidoscopy rate ratio, were too complex to compute the variance directly. A description of how we validated our simulation is presented in eMethods 4 in Supplement 1.

We used the *z* test to compare colonoscopy screening effectiveness (rate ratios and additional benefits) in women vs men. All tests were 2-sided and statistical significance was defined as *P* < .05. Rates are presented per 100 000 person-years. The primary analysis of the study was conducted from January 19 to December 30, 2021, using Stata, version 17.0 (StataCorp LLC), and SAS, version 9.4 (SAS Institute Inc).

## Results

### Baseline Characteristics

Our analyses comprised 358 204 individuals aged 55 to 64 years, 181 971 women (51%) with 2 795 577 person-years of follow-up and 176 233 men (49%) with 2 590 547 person-years of follow-up. Baseline characteristics of the 4 trials are shown in eTable 2 in Supplement 1. The median follow-up time of the trials ranged from 15 to 17 years (up to 19 years for mortality). In the 4 trials, screening attendance among individuals randomly assigned to screening was 54% to 85% for women and 62% to 89% for men. Pooled characteristics of follow-up and CRC incidence and mortality are presented in Table 1. The percentage of individuals who underwent colonoscopy following a positive sigmoidoscopy result was lowest in the UKFSST (2% for women, 5% for men) and highest in the PLCO (18% for women, 28% for men).

**Table 1.** Pooled Characteristics of Enrolled Individuals and CRC Incidence and Mortality From 4 Sigmoidoscopy Screening Trials

Characteristic	Total participants, No. (%)	Usual care, participants, No. (%)	Screening, participants, No. (%)		
			Nonattenders	Attenders	
				Colonoscopy^a^	No colonoscopy^a^
No. of individuals					
Women	181 971	112 245	19 058	6138	44 530
Men	176 233	108 466	15 893	9796	42 078
Person-years, CRC incidence					
Women	2 795 577	1 724 973	283 778	91 330	695 497
Men	2 590 547	1 589 544	218 599	142 282	640 122
CRC incidence^b^					
All	8508 (2.4)	5823 (2.6)	935 (2.7)	562 (3.5)	1188 (1.4)
Distal	4912 (1.4)	3526 (1.6)	577 (1.7)	384 (2.4)	425 (0.5)
Proximal	3523 (1.0)	2251 (1.0)	348 (1.0)	171 (1.1)	753 (0.9)
Women					
All	3544 (1.9)	2368 (2.1)	411 (2.2)	180 (2.9)	585 (1.3)
Distal	1789 (1.0)	1260 (1.1)	225 (1.2)	120 (2.0)	184 (0.4)
Proximal	1729 (1.0)	1092 (1.0)	180 (0.9)	60 (1.0)	397 (0.9)
Men					
All	4964 (2.8)	3455 (3.2)	524 (3.3)	382 (3.9)	603 (1.4)
Distal	3123 (1.8)	2266 (2.1)	352 (2.2)	264 (2.7)	241 (0.6)
Proximal	1794 (1.0)	1159 (1.1)	168 (1.1)	111 (1.1)	356 (0.8)
Person-years, CRC mortality					
Women	2 947 166	1 803 074	303 858	101 242	738 991
Men	2 736 393	1 666 686	233 322	157 974	678 411
CRC mortality^b^					
All	2591 (0.7)	1784 (0.8)	328 (0.9)	113 (0.7)	366 (0.4)
Distal	1353 (0.4)	987 (0.4)	205 (0.6)	64 (0.4)	97 (0.1)
Proximal	1089 (0.3)	703 (0.3)	102 (0.3)	45 (0.3)	239 (0.3)
Women					
All	1034 (0.6)	689 (0.6)	137 (0.7)	41 (0.7)	167 (0.4)
Distal	447 (0.2)	310 (0.3)	77 (0.4)	20 (0.3)	40 (0.1)
Proximal	523 (0.3)	338 (0.3)	52 (0.3)	21 (0.3)	112 (0.3)
Men					
All	1557 (0.9)	1095 (1.0)	191 (1.2)	72 (0.7)	199 (0.5)
Distal	906 (0.5)	677 (0.6)	128 (0.8)	44 (0.4)	57 (0.1)
Proximal	566 (0.3)	365 (0.3)	50 (0.3)	24 (0.2)	127 (0.3)

Abbreviation: CRC, colorectal cancer.

^a^ Colonoscopy after positive finding at sigmoidoscopy screening.

^b^ Total number of CRC cases and CRC deaths is greater than the sum of cases by tumor location because of cancer cases with unspecified tumor location.

### Colonoscopy Screening Simulation

#### Colorectal Cancer Incidence

**Figure 2.**
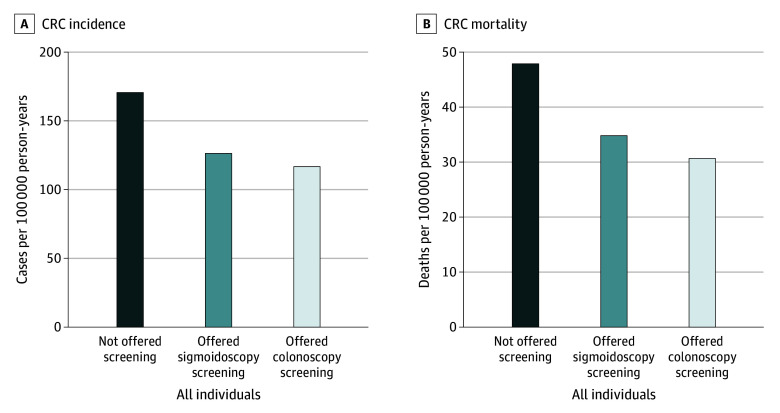
Colorectal Cancer (CRC) Incidence and Mortality Rates After Endoscopic Screening Individuals offered sigmoidoscopy screening were the observed analysis groups, and individuals offered colonoscopy screening were the simulated analysis groups.

For women and men combined, sigmoidoscopy screening prevented an estimated 37 (95% CI, 30-44) CRC cases per 100 000 person-years compared with usual care (Figure 2A, Table 2), corresponding to a 23% (rate ratio, 0.77 [95% CI, 0.74-0.81]) CRC incidence reduction (Table 3). With colonoscopy screening, an estimated 50 (95% CI, 42-58) CRC cases would be prevented per 100 000 person-years, corresponding to 30% (rate ratio, 0.70 [95% CI, 0.66-0.75]) CRC incidence reduction. The additional benefit of colonoscopy screening compared with sigmoidoscopy screening was 12 (95% CI, 10-14) fewer CRC cases per 100 000 person-years, corresponding to 6.9 percentage points (95% CI, 6.0-7.9 percentage points) CRC incidence reduction.

**Table 2.** Numbers of CRC Cases and CRC Deaths Prevented After Sigmoidoscopy or Colonoscopy Screening Compared With Usual Care in Intention-to-Treat Analyses^a^

CRC	Compared with usual care				Colonoscopy screening compared with sigmoidoscopy	
	As observed in sigmoidoscopy trials		As estimated for colonoscopy			
	No. of events prevented (95% CI)	NNS (95% CI)^b^	No. of events prevented (95% CI)	NNS (95% CI)^b^	Additional No. of events prevented (95% CI)	Numbers needed to switch (95% CI)^b^
Incidence						
All individuals	37 (30-44)	188 (158-231)	50 (42-58)	139 (118-164)	12 (10-14)	560 (486-661)
Women	20 (11-28)	352 (243-638)	31 (21-42)	218 (161-336)	11 (8-14)	610 (474-853)
Men	56 (46-67)	126 (106-155)	70 (59-82)	100 (85-120)	13 (10-15)	541 (458-661)
Mortality						
All individuals	11 (7-14)	598 (450-888)	15 (11-19)	415 (327-567)	4 (3-5)	1611 (1275-2188)
Women	4 (0-9)	1478 (733-infinity)	8 (2-13)	807 (478-2572)	3 (1-4)	2248 (1376-6140)
Men	18 (12-23)	367 (280-533)	23 (17-29)	281 (222-383)	5 (4-6)	1300 (1017-1798)

Abbreviations: CRC, colorectal cancer; NNS, number needed to invite to screening.

^a^ Numbers are presented per 100 000 person-years; the number of additional events prevented is not exactly the difference of colonoscopy effectiveness minus sigmoidoscopy effectiveness because the trial weights were slightly different.

^b^ Number of individuals who need to switch screening method (from sigmoidoscopy to colonoscopy) to prevent 1 additional event.

**Table 3.** Results of Intention-to-Treat Analysis on CRC Incidence and Mortality After Sigmoidoscopy or Colonoscopy Screening^a^

CRC	Distal CRC	Proximal CRC		All CRC		
	As observed in sigmoidoscopy trials, rate ratio (95% CI)	As observed in sigmoidoscopy trials, rate ratio (95% CI)	As estimated for colonoscopy, rate ratio (95% CI)	As observed in sigmoidoscopy trials, rate ratio (95% CI)	As estimated for colonoscopy, rate ratio (95% CI)	Colonoscopy screening compared with sigmoidoscopy, additional reduction (95% CI)^b^
Incidence						
All individuals	0.66 (0.62-0.71)	0.94 (0.88-1.01)	0.76 (0.70-0.83)	0.77 (0.74-0.81)	0.70 (0.66-0.75)	6.9 (6.0-7.9)
Women	0.72 (0.65-0.80)	1.00 (0.90-1.10)	0.84 (0.74-0.94)	0.85 (0.79-0.91)	0.77 (0.70-0.85)	8.4 (6.2-10.5)
Men	0.64 (0.59-0.69)	0.90 (0.81-0.99)	0.71 (0.64-0.79)	0.72 (0.68-0.77)	0.66 (0.62-0.72)	5.8 (4.8-6.9)
Mortality						
All individuals	0.64 (0.57-0.72)	0.92 (0.81-1.05)	0.70 (0.60-0.81)	0.76 (0.70-0.83)	0.68 (0.61-0.76)	7.6 (5.7-9.6)
Women	0.79 (0.64-0.97)	0.94 (0.78-1.13)	0.79 (0.64-0.98)	0.87 (0.76-0.99)	0.80 (0.67-0.96)	7.8 (3.1-12.5)
Men	0.58 (0.49-0.67)	0.91 (0.76-1.08)	0.66 (0.54-0.80)	0.70 (0.63-0.78)	0.62 (0.54-0.71)	7.3 (5.3-9.4)

Abbreviation: CRC, colorectal cancer.

^a^ Sigmoidoscopy screening effects are estimated from pooled data from the 4 randomized sigmoidoscopy screening trials. Colonoscopy screening effects are estimated from the same data, but with the screening effect in the proximal colon (by colonoscopy) assumed to be similar to the sigmoidoscopy screening effect in the distal colorectum.

^b^ Numbers are percentage points.

Estimated rate ratios for CRC by colonoscopy screening were 0.77 (95% CI, 0.70-0.85) in women and 0.66 (95% CI, 0.62-0.72) in men (*P* = .02 for sex difference) (Table 3), corresponding to additional benefits of 11 (95% CI, 8-14) fewer CRC cases per 100 000 person-years in women and 13 (95% CI, 10-15) in men following colonoscopy compared with sigmoidoscopy screening (*P* = .33 for sex difference) (Table 2). Results for each trial are presented in eTable 3 in Supplement 1.

The NNS with sigmoidoscopy compared with usual care to prevent 1 CRC was 188 (95% CI, 158-231) for women and men combined, 352 (95% CI, 243-638) for women, and 126 (95% CI, 106-155) for men (Table 2). The NNS with colonoscopy compared with usual care to prevent 1 CRC was 139 (95% CI, 118-164) for women and men combined, 218 (95% CI, 161-336) for women, and 100 (95% CI, 85-120) for men. The number needed to switch from sigmoidoscopy to colonoscopy screening to prevent 1 additional CRC was 560 (95% CI, 486-661) for women and men combined, 610 (95% CI, 474-853) for women, and 541 (95% CI, 458-661) for men. Results of our validation of the CRC incidence simulation, using 10-year data from the NORCCAP and NordICC trials, are presented in eAppendix 2 in Supplement 1.

#### Colorectal Cancer Mortality

For women and men combined, sigmoidoscopy screening prevented 11 (95% CI, 7-14) CRC deaths per 100 000 person-years compared with usual care (Figure 2B, Table 2), corresponding to a 24% (rate ratio, 0.76 [95% CI, 0.70-0.83]) CRC mortality reduction (Table 3). With colonoscopy screening, 15 (95% CI, 11-19) CRC deaths would be prevented per 100 000 person-years, corresponding to 32% (rate ratio, 0.68 [95% CI, 0.61-0.76]) CRC mortality reduction. The additional benefit of colonoscopy screening compared with sigmoidoscopy screening was 4 (95% CI, 3-5) CRC deaths per 100 000 person-years, corresponding to 7.6 percentage points (95% CI, 5.7-9.6 percentage points) in CRC mortality reduction.

Estimated rate ratios for colonoscopy screening compared with usual care were 0.80 (95% CI, 0.67-0.96) in women and 0.62 (95% CI, 0.54-0.71) in men (*P* = .02 for sex difference) (Table 3). Compared with sigmoidoscopy screening, colonoscopy provided additional benefits of 3 (95% CI, 1-4) fewer CRC deaths per 100 000 person-years in women and 5 (95% CI, 4-6) fewer in men (*P* = .04 for sex difference) (Table 2). Results for each trial are presented in eTable 3 in Supplement 1.

The NNS with sigmoidoscopy compared with usual care to prevent 1 CRC death was 598 (95% CI, 450-888) for women and men combined, 1478 (95% CI, 733-infinity) for women and 367 (95% CI, 280-533) for men (Table 2). The NNS with colonoscopy compared with usual care to prevent 1 CRC death was 415 (95% CI, 327-567) for women and men combined, 807 (95% CI, 478-2572) for women, and 281 (95% CI, 222-383) for men. The number needed to switch from sigmoidoscopy screening to colonoscopy to prevent 1 additional CRC death was 1611 (95% CI, 1275-2188) for women and men combined, 2248 (95% CI, 1376-6140) for women, and 1300 (95% CI, 1017-1798) for men.

## Discussion

Our simulation analysis in this comparative effectiveness study found that the 15-year effectiveness of colonoscopy screening compared with usual care would be a 30% reduction in CRC incidence and a 32% reduction in CRC mortality. Compared with sigmoidoscopy screening, colonoscopy screening provided 7 percentage points’ additional reductions in CRC incidence and mortality. The additional preventive effect of colonoscopy, compared with sigmoidoscopy, was less than what was achieved by introducing sigmoidoscopy screening where no screening exists. Our findings were based on screening with a single examination and did not include information on additional adverse effects or harms of colonoscopy, which is needed to comprehensively assess the benefit-to-harm ratio of different screening modalities.

The validation of our simulation model showed that the benefit of colonoscopy was comparable to the observed effect in the Norwegian portion of NordICC.^11^ Because no country-specific data on CRC mortality were available for NordICC, we could not perform a similar validation for mortality. Colonoscopy did not have a statistically significant effect on CRC mortality in the NordICC trial, whereas all sigmoidoscopy screening trials except SCORE found a reduction in CRC mortality after 10 years of follow-up.^11,14,15,16,17^ Insufficient statistical power in the NordICC trial has been suggested as a possible explanation for this observation, but improved treatment may also contribute.^18^ Long-term follow-up data of the NordICC trial will shed further light on the effectiveness of colonoscopy screening for CRC mortality.

Currently, 1 trial has reported data on the effect of colonoscopy screening on CRC incidence and mortality,^11^ but results from 3 other trials are awaited.^19,20,21^ Even before results from any randomized clinical trial were available, several health systems propagated colonoscopy screening under the assumption that the effect on CRC incidence and mortality is superior to other screening alternatives.^22,23,24^ Compared with sigmoidoscopy and fecal testing, colonoscopy involves a higher burden associated with the procedure (eg, more extensive bowel preparation) and occupies more time and resources, which are already limited.^25^ In meta-analyses, bleeding occurs in 5 per 1000 colonoscopies and perforation in 0.5 per 1000 colonoscopies; corresponding numbers from the sigmoidoscopy trials were 0.3 per 1000 screening attenders for both bleeding and perforation.^26,27^ Nongastrointestinal adverse events, such as cardiac events, may also occur after colonoscopy.^28,29^

The estimated reduction in CRC incidence and mortality from colonoscopy screening appeared to be larger in men than women, which is in line with a recent finding by members of our team of a possible sex difference in the estimated effectiveness of sigmoidoscopy screening.^30^ That study and other previous studies found a higher proportion of proximal cancers among women,^31^ who seem to have a higher risk of advanced adenomas and cancer in the proximal colon, compared with the distal colorectum.^32,33^ More than half of women with an advanced adenoma located in the proximal colon may not have any lesions in the distal part of the colorectum,^34,35^ which may explain the sex difference in benefit from sigmoidoscopy screening, in which the proximal colon is not reached.^36^ Indeed, our results showed that more men than women were referred to colonoscopy after sigmoidoscopy screening.

We used screening adherence as observed in the sigmoidoscopy screening trials, thus resembling an intention-to-treat analysis. This may explain the slightly lower effectiveness (50 fewer CRC cases per 100 000 person-years) compared with a previous modeling study (66 fewer CRC cases per 100 000 person-years) in which adherence was assumed to be 100%.^37^ In addition, colonoscopy screening trials have shown lower attendance rates (eg, 42% in the NordICC trial^11^) than in our simulation. A lower attendance rate would attenuate our estimates of an additional benefit by switching to colonoscopy screening.

### Strengths and Limitations

The main strength of this study is the use of observed, long-term follow-up outcomes from all randomized sigmoidoscopy screening trials. Data from each trial had identical definitions of proximal and distal CRC, which reduced heterogeneity. We also validated our simulation method using observed results from the NordICC colonoscopy screening trial.^11^

Our study has limitations. We did not consider additional adverse effects or harms of colonoscopy. Furthermore, our assumptions may be criticized. First, the assumption that attendance rate for colonoscopy screening would be similar to sigmoidoscopy screening was probably too optimistic. Indeed, attendance rates in colonoscopy screening trials have been lower than in trials of other CRC screening methods.^11,19,38^ Our assumption may thus overestimate the additional benefit of colonoscopy screening. Second, we assumed that a colonoscopy after a positive sigmoidoscopy screening had the same sensitivity for adenomas and cancer (eg, similar effectiveness in CRC incidence and mortality reduction) in the distal and proximal colon as a screening colonoscopy. However, colonoscopies performed after a positive screening test may be more meticulous when the patient has a high pretest probability of having pathological findings.^39,40^ Furthermore, we assumed that sigmoidoscopy and colonoscopy had similar effectiveness for CRC incidence and mortality reduction in the distal colon, but because of more thorough bowel preparation, more lesions may be detected by colonoscopy.^39,41,42^ However, only 0.5% of individuals with negative sigmoidoscopy screening results were diagnosed with distal CRC during follow-up, suggesting that few distal CRCs remained undetected after a negative sigmoidoscopy result. Baseline data from the NordICC trial indicate only a slightly higher adenoma detection rate in the distal colon with colonoscopy compared with sigmoidoscopy screening.^43,44^ In observational studies, the added benefit of colonoscopy compared with sigmoidoscopy screening was confined to proximal colon cancer incidence and mortality.^38,45^

Our assumption that the effectiveness of colonoscopy screening in the proximal colon is similar to the effectiveness of sigmoidoscopy screening in the distal colon likely overestimates the effectiveness of colonoscopy screening. In 1 study, the simulated 10-year effectiveness of colonoscopy screening was similar to NordICC when the effectiveness in the proximal colon was reduced to 70% of the effectiveness in the distal colon.^46^ Possible reasons for this finding are a higher prevalence of serrated polyps in the proximal compared with the distal colon, and greater difficulty in the detection of preneoplastic lesions in the proximal colon.^10,47^ The incidence of proximal adenomas and cancers also shows an increasing trend with age.^48^ Furthermore, compared with men, women appear to have a higher proportion of advanced adenoma and cancer in the proximal colon.^32,33^ Overall, our assumptions likely entailed overestimation of the benefit of colonoscopy screening and hence provide an upper limit of what may be achieved with colonoscopy compared with sigmoidoscopy.

## Conclusions

In this comparative effectiveness study of CRC screening, colonoscopy screening was estimated to reduce CRC incidence by 30% and CRC mortality by 32%, compared with usual care. Compared with sigmoidoscopy screening, colonoscopy screening provided 6.9 percentage points additional reductions in CRC incidence and 7.6 percentage points additional reductions in CRC mortality, which means that the additional preventive effect of colonoscopy compared with sigmoidoscopy was less than what was achieved by introducing sigmoidoscopy screening where no screening existed.

## Notes

Supplement 1. **eTable 1.** Trial definition of a positive sigmoidoscopy screening test **eTable 2.** Characteristics of the four sigmoidoscopy screening trials **eTable 3.** Results of intention to treat analysis on CRC incidence and mortality after sigmoidoscopy or colonoscopy screening in each trial **eMethods 1.** Summary description of included endoscopy screening trials **eMethods 2.** Details on data acquisition and data protection legislation **eMethods 3.** Presentation of calculations to estimate screening effect on CRC incidence and mortality **eMethods 4.** Method description, validation of the simulation study **eAppendix 1.** International Society for Pharmacoeconomics and Outcomes Research (ISPOR) reporting guideline checklist **eAppendix 2.** Results validation of the simulation study, colorectal cancer incidence

Supplement 2.
Data Sharing Statement

## References

[1] Lauby-Secretan B, Vilahur N, Bianchini F, Guha N, Straif K; International Agency for Research on Cancer Handbook Working Group. The IARC perspective on colorectal cancer screening. N Engl J Med. 2018;378(18):1734-1740. doi:10.1056/NEJMsr1714643 29580179 PMC6709879

[2] Davidson KW, Barry MJ, Mangione CM, ; US Preventive Services Task Force. Screening for colorectal cancer: US Preventive Services Task Force recommendation statement. JAMA. 2021;325(19):1965-1977. doi:10.1001/jama.2021.6238 34003218

[3] Holme Ø, Løberg M, Kalager M, ; NORCCAP Study Group. Long-term effectiveness of sigmoidoscopy screening on colorectal cancer incidence and mortality in women and men: a randomized trial. Ann Intern Med. 2018;168(11):775-782. doi:10.7326/M17-1441 29710125 PMC6853067

[4] Atkin W, Wooldrage K, Parkin DM, . Long term effects of once-only flexible sigmoidoscopy screening after 17 years of follow-up: the UK Flexible Sigmoidoscopy Screening randomised controlled trial. Lancet. 2017;389(10076):1299-1311. doi:10.1016/S0140-6736(17)30396-3 28236467 PMC6168937

[5] Miller EA, Pinsky PF, Schoen RE, Prorok PC, Church TR. Effect of flexible sigmoidoscopy screening on colorectal cancer incidence and mortality: long-term follow-up of the randomised US PLCO cancer screening trial. Lancet Gastroenterol Hepatol. 2019;4(2):101-110. doi:10.1016/S2468-1253(18)30358-3 30502933 PMC6335177

[6] Senore C, Riggi E, Armaroli P, ; SCORE Working Group. Long-term follow-up of the Italian Flexible Sigmoidoscopy Screening Trial. Ann Intern Med. 2022;175(1):36-45. doi:10.7326/M21-0977 34748376

[7] Shergill AK, Conners EE, McQuaid KR, . Protective association of colonoscopy against proximal and distal colon cancer and patterns in interval cancer. Gastrointest Endosc. 2015;82(3):529-37.e1. doi:10.1016/j.gie.2015.01.053 25936449 PMC4540647

[8] Qaseem A, Crandall CJ, Mustafa RA, ; Clinical Guidelines Committee of the American College of Physicians. Screening for colorectal cancer in asymptomatic average-risk adults: a guidance statement from the American College of Physicians. Ann Intern Med. 2019;171(9):643-654. doi:10.7326/M19-0642 31683290 PMC8152103

[9] Brenner H, Chang-Claude J, Seiler CM, Rickert A, Hoffmeister M. Protection from colorectal cancer after colonoscopy: a population-based, case-control study. Ann Intern Med. 2011;154(1):22-30. doi:10.7326/0003-4819-154-1-201101040-00004 21200035

[10] Kahi CJ, Pohl H, Myers LJ, Mobarek D, Robertson DJ, Imperiale TF. Colonoscopy and colorectal cancer mortality in the Veterans Affairs Health Care System: a case-control study. Ann Intern Med. 2018;168(7):481-488. doi:10.7326/M17-0723 29532085

[11] Bretthauer M, Løberg M, Wieszczy P, ; NordICC Study Group. Effect of colonoscopy screening on risks of colorectal cancer and related death. N Engl J Med. 2022;387(17):1547-1556. doi:10.1056/NEJMoa2208375 36214590

[12] Dominitz JA, Robertson DJ. Understanding the results of a randomized trial of screening colonoscopy. N Engl J Med. 2022;387(17):1609-1611. doi:10.1056/NEJMe2211595 36214591

[13] Berger ML, Mamdani M, Atkins D, Johnson ML. Good research practices for comparative effectiveness research: defining, reporting and interpreting nonrandomized studies of treatment effects using secondary data sources: the ISPOR Good Research Practices for Retrospective Database Analysis Task Force Report—Part I. Value Health. 2009;12(8):1044-1052. doi:10.1111/j.1524-4733.2009.00600.x 19793072

[14] Holme Ø, Løberg M, Kalager M, . Effect of flexible sigmoidoscopy screening on colorectal cancer incidence and mortality: a randomized clinical trial. JAMA. 2014;312(6):606-615. doi:10.1001/jama.2014.8266 25117129 PMC4495882

[15] Schoen RE, Pinsky PF, Weissfeld JL, ; PLCO Project Team. Colorectal-cancer incidence and mortality with screening flexible sigmoidoscopy. N Engl J Med. 2012;366(25):2345-2357. doi:10.1056/NEJMoa1114635 22612596 PMC3641846

[16] Atkin WS, Edwards R, Kralj-Hans I, ; UK Flexible Sigmoidoscopy Trial Investigators. Once-only flexible sigmoidoscopy screening in prevention of colorectal cancer: a multicentre randomised controlled trial. Lancet. 2010;375(9726):1624-1633. doi:10.1016/S0140-6736(10)60551-X 20430429

[17] Segnan N, Armaroli P, Bonelli L, ; SCORE Working Group. Once-only sigmoidoscopy in colorectal cancer screening: follow-up findings of the Italian Randomized Controlled Trial—SCORE. J Natl Cancer Inst. 2011;103(17):1310-1322. doi:10.1093/jnci/djr284 21852264

[18] Hanley JA. Colonoscopy screening and colorectal cancer incidence and mortality. N Engl J Med. 2023;388(4):376-379. doi:10.1056/NEJMc2215192 36720141

[19] Castells A, Quintero E. Programmatic screening for colorectal cancer: the COLONPREV study. Dig Dis Sci. 2015;60(3):672-680. doi:10.1007/s10620-014-3446-2 25492501

[20] Fritzell K, Forsberg A, Wangmar J, Wengström Y, Bottai M, Hultcrantz R. Gender, having a positive FIT and type of hospital are important factors for colonoscopy experience in colorectal cancer screening—findings from the SCREESCO study. Scand J Gastroenterol. 2020;55(11):1354-1362. doi:10.1080/00365521.2020.1820568 32946700

[21] Dominitz JA, Robertson DJ, Ahnen DJ, . Colonoscopy vs. Fecal Immunochemical Test in Reducing Mortality From Colorectal Cancer (CONFIRM): rationale for study design. Am J Gastroenterol. 2017;112(11):1736-1746. doi:10.1038/ajg.2017.286 29016565

[22] Bénard F, Barkun AN, Martel M, von Renteln D. Systematic review of colorectal cancer screening guidelines for average-risk adults: summarizing the current global recommendations. World J Gastroenterol. 2018;24(1):124-138. doi:10.3748/wjg.v24.i1.124 29358889 PMC5757117

[23] Ladabaum U, Dominitz JA, Kahi C, Schoen RE. Strategies for colorectal cancer screening. Gastroenterology. 2020;158(2):418-432. doi:10.1053/j.gastro.2019.06.043 31394083

[24] Lin JS, Perdue LA, Henrikson NB, Bean SI, Blasi PR. Screening for colorectal cancer: updated evidence report and systematic review for the US Preventive Services Task Force. JAMA. 2021;325(19):1978-1998. doi:10.1001/jama.2021.4417 34003220 PMC13509038

[25] Sharaf RN, Ladabaum U. Comparative effectiveness and cost-effectiveness of screening colonoscopy vs. sigmoidoscopy and alternative strategies. Am J Gastroenterol. 2013;108(1):120-132. doi:10.1038/ajg.2012.380 23247579

[26] Reumkens A, Rondagh EJ, Bakker CM, Winkens B, Masclee AA, Sanduleanu S. Post-colonoscopy complications: a systematic review, time trends, and meta-analysis of population-based studies. Am J Gastroenterol. 2016;111(8):1092-1101. doi:10.1038/ajg.2016.234 27296945

[27] Jodal HC, Helsingen LM, Anderson JC, Lytvyn L, Vandvik PO, Emilsson L. Colorectal cancer screening with faecal testing, sigmoidoscopy or colonoscopy: a systematic review and network meta-analysis. BMJ Open. 2019;9(10):e032773. doi:10.1136/bmjopen-2019-032773 31578199 PMC6797379

[28] Ladabaum U, Mannalithara A, Desai M, Sehgal M, Singh G. Age-specific rates and time-courses of gastrointestinal and nongastrointestinal complications associated with screening/surveillance colonoscopy. Am J Gastroenterol. 2021;116(12):2430-2445. doi:10.14309/ajg.0000000000001531 34693917

[29] Johnson DA, Lieberman D, Inadomi JM, . Increased post-procedural non-gastrointestinal adverse events after outpatient colonoscopy in high-risk patients. Clin Gastroenterol Hepatol. 2017;15(6):883-891.e9. doi:10.1016/j.cgh.2016.12.015 28017846

[30] Juul FE, Cross JA, Schoen RN, et al. 15-Year benefits of sigmoidoscopy screening on colorectal cancer incidence and mortality: a pooled analysis of randomized trials. Ann Intern Med. 2022;175(11):1525-1533. doi:10.7326/M22-0835 36215714

[31] White A, Ironmonger L, Steele RJC, Ormiston-Smith N, Crawford C, Seims A. A review of sex-related differences in colorectal cancer incidence, screening uptake, routes to diagnosis, cancer stage and survival in the UK. BMC Cancer. 2018;18(1):906. doi:10.1186/s12885-018-4786-7 30236083 PMC6149054

[32] Qumseya BJ, Coe S, Wallace MB. The effect of polyp location and patient gender on the presence of dysplasia in colonic polyps. Clin Transl Gastroenterol. 2012;3(7):e20. doi:10.1038/ctg.2012.14 23238292 PMC3412677

[33] Siegel RL, Ward EM, Jemal A. Trends in colorectal cancer incidence rates in the United States by tumor location and stage, 1992-2008. Cancer Epidemiol Biomarkers Prev. 2012;21(3):411-416. doi:10.1158/1055-9965.EPI-11-1020 22219318

[34] Forsberg AM, Kjellström L, Agréus L, . Prevalence of colonic neoplasia and advanced lesions in the normal population: a prospective population-based colonoscopy study. Scand J Gastroenterol. 2012;47(2):184-190. doi:10.3109/00365521.2011.647062 22229966

[35] Schoenfeld P, Cash B, Flood A, ; CONCeRN Study Investigators. Colonoscopic screening of average-risk women for colorectal neoplasia. N Engl J Med. 2005;352(20):2061-2068. doi:10.1056/NEJMoa042990 15901859

[36] McCashland TM, Brand R, Lyden E, de Garmo P; CORI Research Project. Gender differences in colorectal polyps and tumors. Am J Gastroenterol. 2001;96(3):882-886. doi:10.1111/j.1572-0241.2001.03638.x 11280569

[37] Buskermolen M, Cenin DR, Helsingen LM, . Colorectal cancer screening with faecal immunochemical testing, sigmoidoscopy or colonoscopy: a microsimulation modelling study. BMJ. 2019;367:l5383. doi:10.1136/bmj.l5383 31578177 PMC6774435

[38] Segnan N, Senore C, Andreoni B, ; SCORE3 Working Group—Italy. Comparing attendance and detection rate of colonoscopy with sigmoidoscopy and FIT for colorectal cancer screening. Gastroenterology. 2007;132(7):2304-2312. doi:10.1053/j.gastro.2007.03.030 17570205

[39] Schoen RE, Pinsky PF, Weissfeld JL, . Colorectal cancers not detected by screening flexible sigmoidoscopy in the Prostate, Lung, Colorectal, and Ovarian Cancer Screening Trial. Gastrointest Endosc. 2012;75(3):612-620. doi:10.1016/j.gie.2011.10.024 22341106

[40] Wisse PHA, Erler NS, de Boer SY, . Adenoma detection rate and risk for interval postcolonoscopy colorectal cancer in fecal immunochemical test-based screening: a population-based cohort study. Ann Intern Med. 2022;175(10):1366-1373. doi:10.7326/M22-0301 36162114

[41] Saltzman JR, Cash BD, Pasha SF, ; ASGE Standards of Practice Committee. Bowel preparation before colonoscopy. Gastrointest Endosc. 2015;81(4):781-794. doi:10.1016/j.gie.2014.09.048 25595062

[42] Atkin WS, Hart A, Edwards R, . Single blind, randomised trial of efficacy and acceptability of oral picolax versus self administered phosphate enema in bowel preparation for flexible sigmoidoscopy screening. BMJ. 2000;320(7248):1504-1508. doi:10.1136/bmj.320.7248.1504 10834891 PMC27392

[43] Gondal G, Grotmol T, Hofstad B, Bretthauer M, Eide TJ, Hoff G. The Norwegian Colorectal Cancer Prevention (NORCCAP) screening study: baseline findings and implementations for clinical work-up in age groups 50-64 years. Scand J Gastroenterol. 2003;38(6):635-642. doi:10.1080/00365520310003002 12825872

[44] Bretthauer M, Kaminski MF, Løberg M, ; Nordic-European Initiative on Colorectal Cancer (NordICC) Study Group. Population-based colonoscopy screening for colorectal cancer: a randomized clinical trial. JAMA Intern Med. 2016;176(7):894-902. doi:10.1001/jamainternmed.2016.0960 27214731 PMC5333856

[45] Brenner H, Stock C, Hoffmeister M. Effect of screening sigmoidoscopy and screening colonoscopy on colorectal cancer incidence and mortality: systematic review and meta-analysis of randomised controlled trials and observational studies. BMJ. 2014;348:g2467. doi:10.1136/bmj.g2467 24922745 PMC3980789

[46] Jeon J, Meza R, Hazelton WD, Renehan AG, Luebeck EG. Incremental benefits of screening colonoscopy over sigmoidoscopy in average-risk populations: a model-driven analysis. Cancer Causes Control. 2015;26(6):859-870. doi:10.1007/s10552-015-0559-7 25783458 PMC4646080

[47] Hetzel JT, Huang CS, Coukos JA, . Variation in the detection of serrated polyps in an average risk colorectal cancer screening cohort. Am J Gastroenterol. 2010;105(12):2656-2664. doi:10.1038/ajg.2010.315 20717107

[48] Caldarella A, Crocetti E, Messerini L, Paci E. Trends in colorectal incidence by anatomic subsite from 1985 to 2005: a population-based study. Int J Colorectal Dis. 2013;28(5):637-641. doi:10.1007/s00384-013-1672-2 23478843

