# Supplementary material for: Effectiveness of Colonoscopy Screening vs Sigmoidoscopy Screening in Colorectal Cancer
Source: JAMA Netw Open. 2024 Feb 29;7(2):e240007. doi: 10.1001/jamanetworkopen.2024.0007 (PMC10905314; doi:10.1001/jamanetworkopen.2024.0007)
Supplement: Supplement 1. — eTable 1. Trial definition of a positive sigmoidoscopy screening test eTable 2. Characteristics of the four sigmoidoscopy screening trials eTable 3. Results of intention to treat analysis on CRC incidence and mortality after sigmoidoscopy or colonoscopy screening in each trial eMethods 1. Summary description of included endoscopy screening trials eMethods 2. Details on data acquisition and data protection legislation eMethods 3. Presentation of calculations to estimate screening effect on CRC incidence and mortality eMethods 4. Method description, validation of the simulation study eAppendix 1. International Society for Pharmacoeconomics and Outcomes Research (ISPOR) reporting guideline checklist eAppendix 2. Results validation of the simulation study, colorectal cancer incidence [file jamanetwopen-e240007-s001.pdf]

Supplementary Online Content

Juul FE, Cross AJ, Schoen RE, et al. Effectiveness of colonoscopy screening vs sigmoidoscopy screening in colorectal cancer. *JAMA Netw Open*. 2024;7(2):e240007. doi:10.1001/jamanetworkopen.2024.0007

eTable 1. Trial definition of a positive sigmoidoscopy screening test
eTable 2. Characteristics of the four sigmoidoscopy screening trials
eTable 3. Results of intention to treat analysis on CRC incidence and mortality after sigmoidoscopy or colonoscopy screening in each trial
eMethods 1. Summary description of included endoscopy screening trials
e**Methods 2.** Details on data acquisition and data protection legislation
eMethods 3. Presentation of calculations to estimate screening effect on CRC incidence and mortality
eMethods 4. Method description, validation of the simulation study
e**Appendix 1.** International Society for Pharmacoeconomics and Outcomes Research (ISPOR) reporting guideline checklist
eAppendix 2. Results validation of the simulation study, colorectal cancer incidence
eReferences.

This supplementary material has been provided by the authors to give readers additional information about their work.

© 2024 Juul FE et al. *JAMA Network Open*.

eTable 1. Trial definition of a positive sigmoidoscopy screening test

Trial name	Findings at screening defined as a positive test
NORCCAP	Colorectal cancer<br>Any polyp 10 mm or larger in diameter<br>Any adenoma<br>A positive faecal occult blood test*
PLCO	Colorectal cancer<br>Any polyp or mass
UKFSST	Colorectal cancer<br>Any polyp 10 mm or larger in diameter<br>Three or more tubular adenomas smaller than 10 mm in diameter with low-grade dysplasia<br>Any adenoma smaller than 10 mm in diameter with tubulovillous or villous features or high-grade dysplasia
SCORE	Colorectal cancer<br>Any polyp larger than 5 mm in diameter<br>Three or more tubular adenomas 5 mm in diameter or smaller with low-grade dysplasia<br>Any adenoma 5 mm in diameter or smaller with tubulovillous or villous features, or high-grade dysplasia<br>Inadequate bowel preparation and at least one polyp

** Half of individuals randomised to sigmoidoscopy screening were asked to deliver a stool sample for one-time faecal immunochemical testing on the day of the sigmoidoscopy*

© 2024 Juul FE et al. *JAMA Network Open*.

eTable 2. Characteristics of the four sigmoidoscopy screening trials
Age at enrolment was 55 to 64 years.

**Trial Name**	**NORCCAP***	**PLCO†**	**UKFSST**	**SCORE**
Country	Norway	US	UK	Italy
Randomization and consent procedure	Pre to consent randomization based on national population registry	Expression of interest before randomization	Expression of interest before randomization	Expression of interest before randomization
Inclusion period	1999-2000	1993-2001	1994-1999	1995-1999
Population	General population in the city of Oslo and county of Telemark	General population in the catchment areas of ten screening centres	Persons from selected general practices in catchment area of hospitals in 14 geographical centres who expressed an interest in screening based on a questionnaire	Persons randomly selected from registries at six trial centres were assessed for eligibility and interest in screening
Number of enrolled participants	54 690	99 208	170 034	34 272
Screening / usual care	13 638 / 41 052	49 621 / 49 587	57 098 / 112 936	17 136 / 17 136
Screening attendance	65% §	87%	71%	58%
Colonoscopy attendance‡	14.0%	22.6%	3.6%	4.6%
Follow to up time (median)	15 years	16 years (17 for mortality)	17 years	15 years (19 for mortality)
National screening program during trial screening period	No organized CRC screening and very little opportunistic screening	Opportunistic screening	No organized CRC screening and very little opportunistic screening	Opportunistic screening

**Participants randomized to flexible sigmoidoscopy were re to randomised 1:1 to receive a single faecal occult blood test (FOBT) or no additional testing.*
†Flexible sigmoidoscopy was repeated at three or five years after baseline sigmoidoscopy. Screening attenders in the current study were individuals who were screened at baseline, and/or three or five years after baseline.
‡Proportion of individuals who attended colonoscopy after a positive screening test among individuals invited to sigmoidoscopy screening.
§The NORCCAP trial had pre to consent randomization which might affect attendance rate.

© 2024 Juul FE et al. *JAMA Network Open*.

eTable 3. Results of intention to treat analysis on CRC incidence and mortality after sigmoidoscopy or colonoscopy screening in each trial

A. NORwegian Colorectal CAncer Preventiontrial (NORCCAP)

Trial sigmoidoscopy screening effects are used to estimate the trial's colonoscopy screening effects.

	**Distal colorectal cancer**		**Proximal colon cancer**				**All colorectal cancer**					
	As observed in sigmoidoscopy trials		As observed in sigmoidoscopy trials		As estimated for colonoscopy		As observed in sigmoidoscopy trials		As estimated for colonoscopy			
											Numbers needed to switch*	
	Rate ratio	*CI, 95%*	*Rate ratio*	*CI, 95%*	*Rate ratio*	*CI, 95%*	*Rate ratio*	*CI, 95%*	*Rate ratio*	*CI, 95%*	*Individuals*	*CI, 95%*
**CRC incidence**												
All individuals	0.71	(0.59-0.84)	0.91	(0.75-1.09)	0.76	(0.63-0.93)	0.79	(0.69-0.89)	0.73	(0.62-0.85)	565	(381-1095)
Women	0.82	(0.62-1.07)	1.03	(0.81-1.30)	0.92	(0.67-1.21)	0.93	(0.78-1.11)	0.87	(0.69-1.11)	660	(296-inf.)
Men	0.63	(0.49-0.80)	0.76	(0.57-1.02)	0.64	(0.48-0.87)	0.67	(0.55-0.80)	0.62	(0.51-0.77)	709	(455-1606)
**CRC mortality**												
All individuals	0.92	(0.68-1.24)	0.69	(0.47-0.99)	0.66	(0.44-0.98)	0.82	(0.65-1.02)	0.81	(0.62-1.05)	8950	(1929-inf.)
Women	1.34	(0.84-2.11)	0.76	(0.44-1.24)	0.90	(0.52-1.55)	1.05	(0.75-1.44)	1.12	(0.75-1.67)	-	-
Men	0.71	(0.46-1.05)	0.62	(0.34-1.05)	0.52	(0.30-0.92)	0.66	(0.47-0.90)	0.62	(0.44-0.88)	2566	(1188-inf.)

CI: confidence interval, CRC: colorectal cancer.

**Number of individuals that need to switch screening method (from sigmoidoscopy to colonoscopy) to prevent one event.*

© 2024 Juul FE et al. *JAMA Network Open*.

eTable 3 (cont'd).

B. Prostate, Lung, Colorectal and Ovarian cancerscreening trial (PLCO)

Trial sigmoidoscopy screening effects are used to estimate the trial's colonoscopy screening effects.

	**Distal colorectal cancer**		**Proximal colorectal cancer**				**All colorectal cancer**					
	As observed in sigmoidoscopy trials		As observed in sigmoidoscopy trials		As estimated for colonoscopy		As observed in sigmoidoscopy trials		As estimated for colonoscopy			
											*Numbers needed to switch**	
	Rate ratio	*CI, 95%*	*Rate ratio*	*CI, 95%*	*Rate ratio*	*CI, 95%*	*Rate ratio*	*CI, 95%*	*Rate ratio*	*CI, 95%*	*Individuals*	*CI, 95%*
**CRC incidence**												
All individuals	0.75	(0.66-0.86)	0.94	(0.82-1.08)	0.80	(0.68-0.94)	0.85	(0.77-0.93)	0.78	(0.69-0.88)	831	(582-1448)
Women	0.98	(0.79-1.23)	0.88	(0.72-1.07)	0.87	(0.68-1.11)	0.93	(0.80-1.07)	0.92	(0.75-1.13)	11 902	(896-inf.)
Men	0.64	(0.53-0.76)	1.00	(0.82-1.22)	0.79	(0.63-0.98)	0.79	(0.69-0.90)	0.71	(0.60-0.83)	558	(414-859)
**CRC mortality**												
All individuals	0.62	(0.46-0.82)	1.13	(0.86-1.48)	0.86	(0.63-1.17)	0.88	(0.73-1.05)	0.77	(0.60-0.97)	1719	(1129-3596)
Women	0.81	(0.49-1.33)	1.10	(0.75-1.63)	0.98	(0.62-1.54)	1.04	(0.78-1.39)	0.97	(0.66-1.43)	3849	(1201-inf.)
Men	0.54	(0.37-0.76)	1.15	(0.79-1.69)	0.80	(0.53-1.19)	0.78	(0.61-0.99)	0.66	(0.49-0.88)	1280	(839-2691)

CI: confidence interval, CRC: colorectal cancer.

**Number of individuals that need to switch screening method (from sigmoidoscopy to colonoscopy) to prevent one event.*

© 2024 Juul FE et al. *JAMA Network Open*.

eTable 3 (cont'd).

C. UK Flexible Sigmoidoscopy Screening Trial (UKFSST)

Trial sigmoidoscopy screening effects are used to estimate the trial's colonoscopy screening effects.

	**Distal colorectal cancer**		**Proximal colon cancer**				**All colorectal cancer**					
	As observed in sigmoidoscopy trials		As observed in sigmoidoscopy trials		As estimated for colonoscopy		As observed in sigmoidoscopy trials		As estimated for colonoscopy			
											*Numbers needed to switch**	
	Rate ratio	*CI, 95%*	*Rate ratio*	*CI, 95%*	*Rate ratio*	*CI, 95%*	*Rate ratio*	*CI, 95%*	*Rate ratio*	*CI, 95%*	*Individuals*	*CI, 95%*
**CRC incidence**												
All individuals	0.61	(0.56-0.67)	0.94	(0.85-1.04)	0.71	(0.63-0.79)	0.73	(0.68-0.78)	0.65	(0.59-0.70)	413	(352-501)
Women	0.63	(0.54-0.73)	1.03	(0.89-1.19)	0.77	(0.66-0.91)	0.80	(0.72-0.89)	0.69	(0.60-0.79)	420	(326-588)
Men	0.60	(0.54-0.67)	0.86	(0.75-1.00)	0.66	(0.56-0.76)	0.69	(0.63-0.75)	0.62	(0.56-0.69)	416	(340-537)
**CRC mortality**												
All individuals	0.56	(0.47-0.66)	0.92	(0.77-1.11)	0.65	(0.53-0.80)	0.70	(0.62-0.79)	0.60	(0.51-0.70)	1109	(860-1562)
Women	0.64	(0.48-0.86)	0.88	(0.68-1.13)	0.66	(0.49-0.89)	0.74	(0.62-0.90)	0.64	(0.50-0.83)	1434	(891-3666)
Men	0.52	(0.42-0.65)	0.97	(0.76-1.25)	0.66	(0.50-0.89)	0.67	(0.57-0.79)	0.58	(0.47-0.70)	972	(731-1448)

CI: confidence interval, CRC: colorectal cancer.

**Number of individuals that need to switch screening method (from sigmoidoscopy to colonoscopy) to prevent one event.*

© 2024 Juul FE et al. *JAMA Network Open*.

eTable 3 (cont'd).

D. Screening for Colon REctum trial (SCORE)

Trial sigmoidoscopy screening effects are used to estimate the trial's colonoscopy screening effects.

	**Distal colorectal cancer**		**Proximal colon cancer**				**All colorectal cancer**					
	As observed in sigmoidoscopy trials		As observed in sigmoidoscopy trials		As estimated for colonoscopy		As observed in sigmoidoscopy trials		As estimated for colonoscopy			
											*Numbers needed to switch**	
	Rate ratio	*CI, 95%*	*Rate ratio*	*CI, 95%*	*Rate ratio*	*CI, 95%*	*Rate ratio*	*CI, 95%*	*Rate ratio*	*CI, 95%*	*Individuals*	*CI, 95%*
**CRC incidence**												
All individuals	0.70	(0.58-0.83)	1.03	(0.83-1.28)	0.89	(0.72-1.12)	0.81	(0.71-0.93)	0.76	(0.65-0.89)	781	(528-1501)
Women	0.58	(0.42-0.80)	1.15	(0.82-1.61)	0.94	(0.65-1.36)	0.79	(0.63-0.98)	0.71	(0.55-0.92)	623	(399-1492)
Men	0.77	(0.62-0.97)	0.95	(0.72-1.27)	0.86	(0.65-1.14)	0.83	(0.70-0.99)	0.80	(0.66-0.97)	948	(520-5327)
**CRC mortality**												
All individuals	0.68	(0.49-0.96)	0.82	(0.57-1.18)	0.69	(0.45-1.04)	0.77	(0.61-0.98)	0.72	(0.54-0.95)	1942	(1074-10 122)
Women	0.63	(0.33-1.18)	1.27	(0.68-2.40)	1.04	(0.51-2.13)	0.90	(0.59-1.36)	0.81	(0.49-1.35)	2115	(958-inf.)
Men	0.71	(0.47-1.07)	0.66	(0.42-1.04)	0.56	(0.34-0.93)	0.73	(0.54-0.97)	0.68	(0.48-0.97)	1762	(824-inf.)

CI: confidence interval, CRC: colorectal cancer.

**Number of individuals that need to switch screening method (from sigmoidoscopy to colonoscopy) to prevent one event.*

© 2024 Juul FE et al. *JAMA Network Open*.

eMethods 1. Summary description of included endoscopy screening trials

Data used in our simulation analysis were from four randomized sigmoidoscopy screening trials in Norway (NORwegian Colorectal CAncer Prevention trial; NORCCAP) (ClinicalTrials.gov: NCT00119912), the US (Prostate, Lung, Colorectal and Ovarian cancer screening trial; PLCO) (ClinicalTrials.gov: NCT01696981), the UK (UK Flexible Sigmoidoscopy Screening Trial; UKFSST) (ISRCTN number: 28352761) and Italy (Screening for Colon REctum trial; SCORE) (ISRCTN number: 27814061). Additionaly, we used data from the Norwegian part the Nordic-European Initiative on Colorectal Cancer (NordICC) trial (ClinicalTrials.gov: NCT 00883792) on colonoscopy screening to validate our simulation model. All trials were approved by the ethics committees in the respective countries or regions.

In brief, all trials included individuals aged 55-64 years at enrolment, randomised to once-only sigmoidoscopy screening or usual care (i.e. no screening invitation). The NORCCAP trial also included individuals aged 50-54 years and the PLCO trial included individuals aged 65-74 years (these age groups are not included in the current analysis). Individuals randomised to screening in PLCO were also offered a second sigmoidoscopy three or five years later.[1] In NORCCAP, 50% of individuals randomised to screening were asked to deliver a stool sample for one-time faecal immunochemical testing (FIT) on the day of the sigmoidoscopy. Screening in the trials was performed between 1993 and 2001. The primary endpoints in all trials were CRC incidence and mortality.

Participants in the control groups were not offered any intervention as part of the trials, but received health care, including similar access to cancer screening as the general population in each trial area.

All individuals in NORCCAP, UKFSST and SCORE with a positive sigmoidoscopy screening test were referred for colonoscopy (Figure 1). Individuals in PLCO with a positive screening test were referred to their primary care physician for follow-up. There were some differences between the trials in the definition of a positive screening test (eTable 1 in Supplement).

Details from the NordICC trial have been published previously.[2] In brief, screening-naïve individuals 55-64 years of age in Poland, Norway, Sweden, and the Netherlands were randomised 1:2 to once-only colonoscopy screening or usual care (no screening invitation). Screening was performed between 2009 and 2014 and primary endpoints were CRC incidence and mortality after 10 and 15 years.

© 2024 Juul FE et al. *JAMA Network Open*.

eMethods 2. Details on data acquisition and data protection legislation

Due to new data protection legislation in Europe, individual patient data could not be shared across the trials and data therefore needed to be shared as aggregated anonymized data.

In the data from UKFSST, small numbers (7 or lower) could not be specified due to data protection legislation. Accordingly, the value “0-7” was given for any strata where fewer than eight events occurred. In the analyses, we used the value seven for these strata, which may overestimate the number of events; this applied to the screening and usual care group.

© 2024 Juul FE et al. *JAMA Network Open*.

eMethods 3. Presentation of calculations to estimate screening effect on CRC incidence and mortality

Step 1: Sigmoidoscopy screening effect

Cumulative incidence rates in sigmoidoscopy screening and usual care groups were calculated from the observed data in each trial. Rates were then used to calculate rate ratios (formula 1) and number of prevented events (CRC cases or CRC deaths) per 100 000 person-years (formula 2) by sigmoidoscopy screening compared to usual care.

Analysis groups according to manuscript Figure 1.

- Group [A] Individuals randomised to usual care (controls)
- Group [B] Individuals randomised to sigmoidoscopy screening, but declined to undergo screening (non to attenders)
- Group [C] Individuals randomised to sigmoidoscopy screening, attended screening, and had a positive screening test leading to a subsequent colonoscopy (screening positive)
- Group [D] Individuals randomised to sigmoidoscopy screening, attended screening, but did not have a subsequent colonoscopy (screening negative)

Formula 1

$$Rate\ ratio_{Sigmoidoscopy} = \frac{\frac{Events_{Screening\ group}}{PYr_{Screening\ group}}}{\frac{Events_{Usual\ care\ group}}{PYr_{Usual\ care\ group}}} = \frac{\frac{Events_{Screening\ non-attenders} + Events_{Screening+colonoscopy} + Events\ distal_{Screening\ negative} + Events\ proximal_{Screening\ negative}}{PYr_{Screening\ group}}}{\frac{Events_{Usual\ care\ group}}{PYr_{Usual\ care\ group}}}$$

Formula 2

$$Number\ of\ prevented\ events\ per\ PYr_{Sigmoidoscopy} = \frac{Events_{Screening\ group}}{PYr_{Screening\ group}} - \frac{Events_{Usual\ care\ group}}{PYr_{Usual\ care\ group}}$$

PYr = person-year

Results from the individual trials were then pooled in a meta-analysis (the inverse to variance of the trial specific estimates as weights), to calculate rate ratios and number of prevented events per 100 000 person-years across trials. Variance of the overall estimate was the inverse of the sum of the weights.

Step 2: Colonoscopy screening effect

Next, we wanted to estimate the additional number of events (CRC cases or CRC deaths) prevented with colonoscopy, compared to sigmoidoscopy. Estimates were calculated using the observed number of events in the proximal colon among screening negative (i.e. sigmoidoscopy screening attenders without subsequent colonoscopy, group [D] in manuscript Figure 1) and the sigmoidoscopy screening rate ratios for distal CRC (incidence or mortality). First, we calculated the number of proximal events prevented if colonoscopy had been used in the sigmoidoscopy screening negative individuals in each trial:

Formula 3

$$Proximal\ events\ prevented\ by\ switching\ to\ colonoscopy = Events\ proximal\ colon_{Sigmoidoscopy,\ screening\ negative} * (1 - Rate\ ratio_{Sigmoidoscopy\ distal\ colon})$$

The resulting numbers of events prevented were then subtracted from the observed total number of proximal events in the sigmoidoscopy screening arm to estimate the number of events if colonoscopy was used instead of sigmoidoscopy:

© 2024 Juul FE et al. *JAMA Network Open*.

Formula 4

$$Events\ proximal\ colon_{Colonoscopy,\ screening\ group} = Events\ proximal\ colon_{\ Sigmoidoscopy,\ screening\ group} - Proximal\ events\ prevented\ by\ switching\ to\ colonoscopy$$

We then used the resulting number of events in the colonoscopy screening group in each trial to calculate trial specific rate ratios (formula 5) and number of events prevented per 100 000 person-years (formula 6) by colonoscopy compared to usual care:

Formula 5

$$Rate\ ratio_{colonoscopy} = \frac{\frac{Events_{Screening\ group}}{PYr_{Screening\ group}}}{\frac{Events_{Usual\ care\ group}}{PYr_{Usual\ care\ group}}}$$

$$= \frac{\frac{Events_{Sigmoidoscopy,screening\ non-attenders} + Events_{Sigmoidoscopy\ screening\ attenders\ with\ follow-upcolonoscopy} + Events\ distal_{Sigmoidoscopy\ screening\ negative} + Events\ proximal_{Colonoscopy,\ screening\ group}}{PYr_{Screening\ group}}}{\frac{Events_{Usual\ care\ group}}{PYr_{Usual\ care\ group}}}$$

Formula 6

$$Number\ of\ prevented\ cases\ per\ PYr_{colonoscopy} = \frac{Events_{\ sigmoidoscopy,\ Screening\ group} - Events\ prevented\ proximal_{Colonoscopy,\ screening\ group}}{PYr_{Screening\ group}} - \frac{Events_{Usual\ care\ group}}{PYr_{Usual\ care\ group}}$$

PYr = person-years

Finally, we pooled the results from the individual trials in a meta-analyses (again using the inverse-variance of trial specific estimates as weights) to calculate rate ratios and number of prevented events per 100 000 person-years across trials, variance of the overall estimate being again the inverse of the sum of the weights. Bootstrapping was used to estimate the variance of the colonoscopy metrics.

© 2024 Juul FE et al. *JAMA Network Open.*

eMethods 4. Method description, validation of the simulation study

To validate the results from our simulation, we compared 10-year CRC incidence in the Norwegian sigmoidoscopy trial (NORCCAP) with the 10-year data from the Norwegian part of the NordICC colonoscopy trial.[3,4] Participants in the two trials are comparable; they live in the same country, had the same age, and participation rates for screening were comparable (sigmoidoscopy: 65%, colonoscopy: 61%). Both screening tools were compared with usual care, and post-randomization consent was used in both trials. With a similar analytical approach as previously described (except we used individuals instead of person-years to make it comparable to NordICC), we simulated the colonoscopy screening effect in NORCCAP after 10-years follow-up, and compared the results to the observed results from the NordICC trial.

© 2024 Juul FE et al. *JAMA Network Open*.

eAppendix 1. International Society for Pharmacoeconomics and Outcomes Research (ISPOR) reporting guideline checklist [5]

Section and topic	Item #	Descriptor	Where in manuscript
Study Design	1	Description of Study Design	Manuscript pages 6 and 7.
Introduction<br>• Background	2	Scientific background and explanation of rationale including discussion of the suitability of the database employed	Manuscript pages 6 and 7, eMethods 1.
Methods<br>• Defining the question<br>• Objectives<br>• Selection of study design<br>• Selection of data source<br>○ Definition of treatment cohorts	3	Clearly defined goals of the study with description of specific sub-questions. Description of study design and why it was chosen; Description of strengths and weaknesses of data source and how study groups were identified, including description of critical variables: diagnostic criteria, exposures, and potential confounders	Manuscript pages 6 and 7, eMethods 1-3.
• Measurement of treatment effects<br>○ Classification bias	4	Discuss how treatment effects were measured and how classification bias was addressed.	Manuscript pages 7-10.
• Measurement of outcomes<br>○ Classification bias	5	Discuss how outcomes were measured and how classification bias was addressed.	Manuscript pages 7-10.
• Confounding<br>○ By indication<br>○ Measured vs. unmeasured<br>○ Time dependent<br>• Analytic plan to address confounding	6	Discuss the potential for confounding, both measured and unmeasured, and how this was assessed and addressed	Not discussed in the current study because it is based on randomized controlled trials.
Discussion<br>• Internal validity	7	Interpretation of results, taking into account confounding and imprecision of results	Manuscript pages 14-17, eMethods 1 and 3.
• Generalizability	8	Generalizability (external validity) of the study findings.	Manuscript pages 14-16.
• Overall evidence	9	General interpretation of the results in the context of current evidence.	Manuscript pages 14-17.

© 2024 Juul FE et al. *JAMA Network Open*.

eAppendix 2. Results validation of the simulation study, colorectal cancer incidence

The 10-year risk of CRC was 1.5% in the control groups in both the NORCCAP trial and the Norwegian part of the NordICC trial. In NORCCAP, the estimated effect of colonoscopy after 10-year follow-up was 26 (95% CI, 3-49) fewer CRC cases per 10 000 individuals, corresponding to a risk ratio of 0.83 (95% CI, 0.70-0.99). In comparison, the observed result from the Norwegian part of the NordICC trial was 37 (95% CI, 7-68) fewer CRC cases per 10 000 individuals, corresponding to a risk ratio of 0.76 (95% CI, 0.58-0.94).[4]

© 2024 Juul FE et al. *JAMA Network Open*.

eReferences.

1. Miller EA, Pinsky PF, Schoen RE, Prorok PC, Church TR. Effect of flexible sigmoidoscopy screening on colorectal cancer incidence and mortality: long-term follow-up of the randomised US PLCO cancer screening trial. *Lancet Gastroenterol Hepatol.* 2019;4(2):101-110.
2. Bretthauer M, Kaminski MF, Loberg M, et al. Population-Based Colonoscopy Screening for Colorectal Cancer: A Randomized Clinical Trial. *JAMA Intern Med.* 2016;176(7):894-902.
3. Holme O, Loberg M, Kalager M, et al. Effect of flexible sigmoidoscopy screening on colorectal cancer incidence and mortality: a randomized clinical trial. *JAMA.* 2014;312(6):606-615.
4. Bretthauer M, Loberg M, Wieszczy P, et al. Effect of Colonoscopy Screening on Risks of Colorectal Cancer and Related Death. *N Engl J Med.* 2022.
5. Berger ML, Mamdani M, Atkins D, Johnson ML. Good Research Practices for Comparative Effectiveness Research: Defining, Reporting and Interpreting Nonrandomized Studies of Treatment Effects Using Secondary Data Sources: The ISPOR Good Research Practices for Retrospective Database Analysis Task Force Report—Part I. *Value Health.* 2009;12(8):1044-1052.

© 2024 Juul FE et al. *JAMA Network Open*.
