# Supplementary material for: Effectiveness of Colonoscopy Screening vs Sigmoidoscopy Screening in Colorectal Cancer
Source: JAMA Netw Open. 2024 Feb 29;7(2):e240007. doi: 10.1001/jamanetworkopen.2024.0007 (PMC10905314; doi:10.1001/jamanetworkopen.2024.0007)
Supplement: Supplement 2. — Data Sharing Statement [file jamanetwopen-e240007-s002.pdf]

# Data Sharing Statement

Juul. Effectiveness of Colonoscopy Screening vs Sigmoidoscopy Screening in Colorectal Cancer. *JAMA Netw Open*. Published February 29, 2024.
doi:10.1001/jamanetworkopen.2024.0007

**Data**
**Data available:** No

**Additional Information**
**Explanation for why data not available:** Not allowed due to European data regulations.
